# Controlling *In Planta* Gold Nanoparticle Synthesis and Size for Catalysis

**DOI:** 10.1021/acs.est.4c00266

**Published:** 2024-05-23

**Authors:** Marc Loskarn, Zakuan A. S. Harumain, Jessica A. Dobson, Andrew J. Hunt, Con Robert McElroy, Evaldas Klumbys, Emily Johnston, Juliana Sanchez Alponti, James H. Clark, Frans J. M. Maathuis, Neil C. Bruce, Elizabeth L. Rylott

**Affiliations:** † Centre for Novel Agricultural Products, Department of Biology, University of York, Wentworth Way, York YO10 5DD, U.K.; ‡ Green Chemistry Centre of Excellence, Department of Chemistry, University of York, York YO10 5DD, U.K.; § Department of Biotechnology, Kulliyyah of Science, International Islamic University Malaysia, Kuantan Campus, Kuantan 25200, Malaysia; ∥ Materials Chemistry Research Center (MCRC), Centre of Excellence for Innovation in Chemistry, Department of Chemistry, Faculty of Science, Khon Kaen University, Khon Kaen 40002, Thailand

**Keywords:** gold, *Arabidopsis*, catalysis, nanoparticles, peptides, phytomining, metals

## Abstract

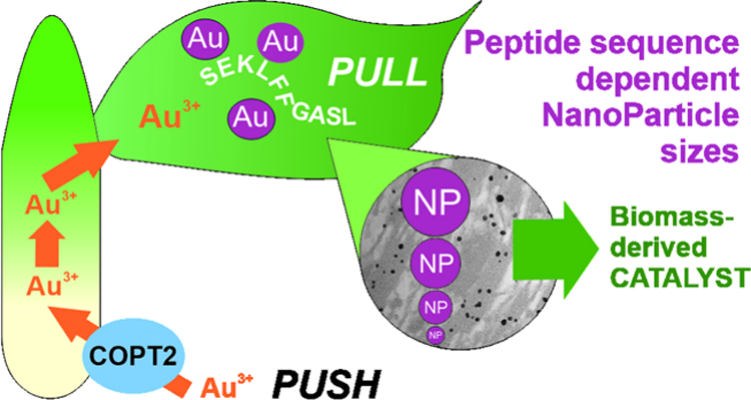

Gold nanoparticles (Au-NPs) are used as catalysts for a diverse range of industrial applications. Currently, Au-NPs are synthesized chemically, but studies have shown that plants fed Au deposit, this element naturally as NPs within their tissues. The resulting plant material can be used to make biomass-derived catalysts. *In vitro* studies have shown that the addition of specific, short (∼10 amino acid) peptide/s to solutions can be used to control the NP size and shape, factors that can be used to optimize catalysts for different processes. Introducing these peptides into the model plant species, *Arabidopsis thaliana* (*Arabidopsis*), allows us to regulate the diameter of nanoparticles within the plant itself, consequently influencing the catalytic performance in the resulting pyrolyzed biomass. Furthermore, we show that overexpressing the copper and gold COPPER TRANSPORTER 2 (COPT2) in *Arabidopsis* increases the uptake of these metals. Adding value to the Au-rich biomass offers the potential to make plant-based remediation and stabilization of mine wastes financially feasible. Thus, this study represents a significant step toward engineering plants for the sustainable recovery of finite and valuable elements from our environment.

## Abstract 2

Our findings are a step closer to developing plants that can recover residual gold from soils contaminated with wastes from traditional mining.

## Introduction

Gold (Au) and platinum group metals (PGMs) are rare elements and are important in an increasing number of biotechnological applications. The chemical properties, and particularly catalytic activities of these metals, mean that suitable substitutes are lacking. However, existing reserves are both dwindling and vulnerable to geopolitically controlled supply restrictions.^1^ These factors mean that it is essential that remaining reserves are not dispersed into the environment but used and recycled responsibly. Mining metals has relatively high environmental impacts per kilogram,^2^ and resultant mine waste areas can be dangerously unstable and leach toxic metals.^3^ One way to capture and concentrate these metals while revegetating and stabilizing waste areas is through phytomining.^4^

Plants dosed with Au, as KAuCl_4_ (or Au^0^, which is then solubilized using cyanide-containing compounds), are known to take up, and deposit, this element within their tissues as nanoparticles (NPs).^5,6^ Many microorganisms also produce metal NPs including Au and PGMs.^7−9^ These bioderived metal NPs have also shown promising catalytic ability in a variety of industrially important reactions such as hydrogenation, Stille, and Suzuki and Heck coupling reactions.^7−9^ Metal NPs are currently synthesized using chemical and physical methods requiring pure starting metals and solvents or harmful chemicals. Using microorganisms to synthesis Au-NPs could potentially be more environmentally friendly and cost-effective.^8^ While microorganisms are already used commercially to recover Au from relatively concentrated wastes such as waste electrical and electronic equipment recycling (WEEE),^10^ plants are better models for mining very dilute concentrations of these valuable metals from larger areas such as mine tailings and road sweepings. In addition, soils in these areas are often degraded and polluted, with a low biodiversity and poor soil structure; plants can remediate soils and restore local ecology.^4^ Using the resulting biomass for catalysis could add further commercial value toward making phytomining commercially viable. To achieve these benefits, plants need to take up and accumulate financially viable levels of the target metal. Plant hyperaccumulators concentrate specific metals including nickel, Cu, and zinc in their tissues to many-fold higher concentrations than those of the surrounding soil.^11^ While Ni hyperaccumulators in the *Odontarrhena* genus (Brassicaceae) are currently used to commercially extract Ni from soils, no hyperaccumulators of Au or PGMs^12^ have yet been identified and are unlikley to occur naturally.

While using plants to extract metals, including Au, from the environment is not new, the costs of growing, harvesting, and transporting the metal-rich plant biomass, in addition to the cost of smelting to the base metal, have been prohibitive to the development of this technology.^13^ Studies have shown that following a low-energy pyrolysis step, the Au- and palladium (Pd)-rich plant biomass can be used directly as a catalyst, potentially negating costs associated with smelting the plant biomass.^6,14,15^ Life-cycle analysis indicates that this method can be more environmentally friendly, with a lower carbon footprint than traditional chemical-based catalyst production.^14,15^ Controlled pyrolysis of the metal NP-containing plant biomass can also be used to enhance the release of specific, value-added platform chemicals such as biofuels from the plant biomass.^15−17^

Brown et al. used an *Escherichia coli*-based, iterative screening approach in which specific, small (∼10 amino acid) peptide sequences, SEKL and GASL, were identified for their ability to reduce and bind Au to form NPs.^18^*In vitro* studies demonstrated that a SEKLGASL peptide seeded the production of Au-NPs with a mean diameter of 180 nm. Sandwiching two phenylalanine (F) residues, with relatively low reducing and binding strengths, between the two SEKL and GASL sequences decreased the NP mean diameter to 80 nm, while replacing the F residues with two tryptophan (T) residues, with relatively high Au reduction and binding strengths, further decreased the resultant NP diameter. Finally, Tan et al.^19^ demonstrated that reversing the SEKL and GASL blocks prevented NP formation.

The application of Au-NPs in catalysis has been extensively studied,^20,21^ and it has been demonstrated that their catalytic properties can be dramatically influenced by the shape and size of the formed particle,^20,22^ with optimal catalysis occurring when the metal NPs used are within the size range of 1–10 nm.^23^ Smaller-sized particles have increased surface area to bulk atom ratios, which allow more surface atoms to be available for catalytic reactions. Thus, in catalytic reactions, smaller NPs are more desirable. However, over repeated cycles of catalysis, NPs coalesce, and the resulting increase in the mean diameter negatively impacts catalytic activity. This agglomeration occurs through Ostwald ripening, where the metal goes into solution, catalyzes the reaction, and then redeposits on the surface. The result is a reduction in the mean size of the NPs, which can also increase the catalyst lifetime.

In this study, we selected specific peptide sequences to express in *Arabidopsis thaliana* (*Arabidopsis*), with the aim of controlling the NP size *in planta* and thus the catalytic properties of the subsequently pyrolyzed plant biomass. To investigate ways to increase Au uptake, we then combined this peptide-based technology with the upregulation of the predominantly root plasma membrane-expressed *Arabidopsis* COPPER TRANSPORTER 2 (COPT2), which in addition to a role in copper (Cu) acquisition and distribution,^24^ also imports Au.^25^

## Materials and Methods

### Production of Transgenic *Arabidopsis* Expressing Short Synthetic Peptides

Transgenes to express peptides producing 180 nm (X-Large-NP), 80 nm (Large-NP), and 40 nm (Medium-NP) mean diameter Au-NP sizes *in vitro*, along with a peptide that inhibited NP production *in vitro* (Small-NP),^19^ were codon-optimized and synthesized for expression in *Arabidopsis*. Flanking regions homologous to the insertion site of the pART7 vector^26^ were added and the oligonucleotides were commercially synthesized. In-Fusion Cloning (Clontech Inc.) was used to introduce the peptide DNA sequence into pART7. The presence of the cassette sequence, containing the peptide-encoding DNA, flanked by the near-constitutively expressing CaMV-*35S* promoter and *ocs* terminator regions, was confirmed using pART7 primers (Table S1) and then transferred into the binary pART27 plasmid using the *Not*I restriction site.^18^ Following transfer into *Agrobacterium tumefaciens*, the pART27-peptide vectors were transformed into *Arabidopsis*, ecotype Col-0, using the floral dip technique.^27^ The pART27 vector contains a selectable marker, *nptII*, which confers resistance to kanamycin. The T1 seeds were screened on half-strength Murashige and Skoog (1/2 MS) agar medium in the presence of 50 μg/mL kanamycin. The T2 lines with kanamycin-resistance segregation ratios indicative of single insertional events were then selected and homozygous T3 lines (judged by 100% resistance toward kanamycin) were selected for further analysis.

### Peptide Expression Analysis

The presence of the peptide-encoding sequences in the transformed lines was confirmed by sequencing, and transcript levels were measured using qPCR. The RNA was extracted from leaves of three-week-old, soil-grown plants using QIAGEN miRNeasy kits. The RNA was polyadenylated, and then cDNA was synthesized using the Agilent Technologies miRNA first-strand cDNA synthesis kit, with the polyA tail extension reverse primer and the forward primers listed in Table S1. qPCR was performed using a SensiFast SYBR No-ROX kit (Bioline) and peptide-encoding transcript levels expressed relative to the *ACTIN2* gene (At3g18780) and the method of Livak.^28^

### Production of Transgenic *Arabidopsis* Expressing COPT2

The *Arabidopsis* COPT2 sequence was artificially codon-optimized (Thermo Fisher Scientific) for overexpression in *Arabidopsis*, and intron sequences were removed to allow transcript quantification of the transgene. The optimized sequence, with 5′ and 3′, respectively, *Xhol* and *Bam*HI sites, was cloned into *pART7* then transferred into the binary pMLBart vector using the *Not*I restriction site.^26^ The pMLBart-COPT2 vector was transformed into *Arabidopsis*, and homozygous T3 lines (judged by 100% resistance toward Basta) were selected for further analysis, as described above.

### COPT2 Expression Analysis

To monitor the expression of COPT2, the RNA was extracted from three-week old rosette leaves and qPCR was performed as described^29^ using the COPT2 primers listed in Table S1, and analyzed as above.

### Production of Transgenic *Arabidopsis* Expressing the Small-NP Peptide and COPT2

**Figure 1:**
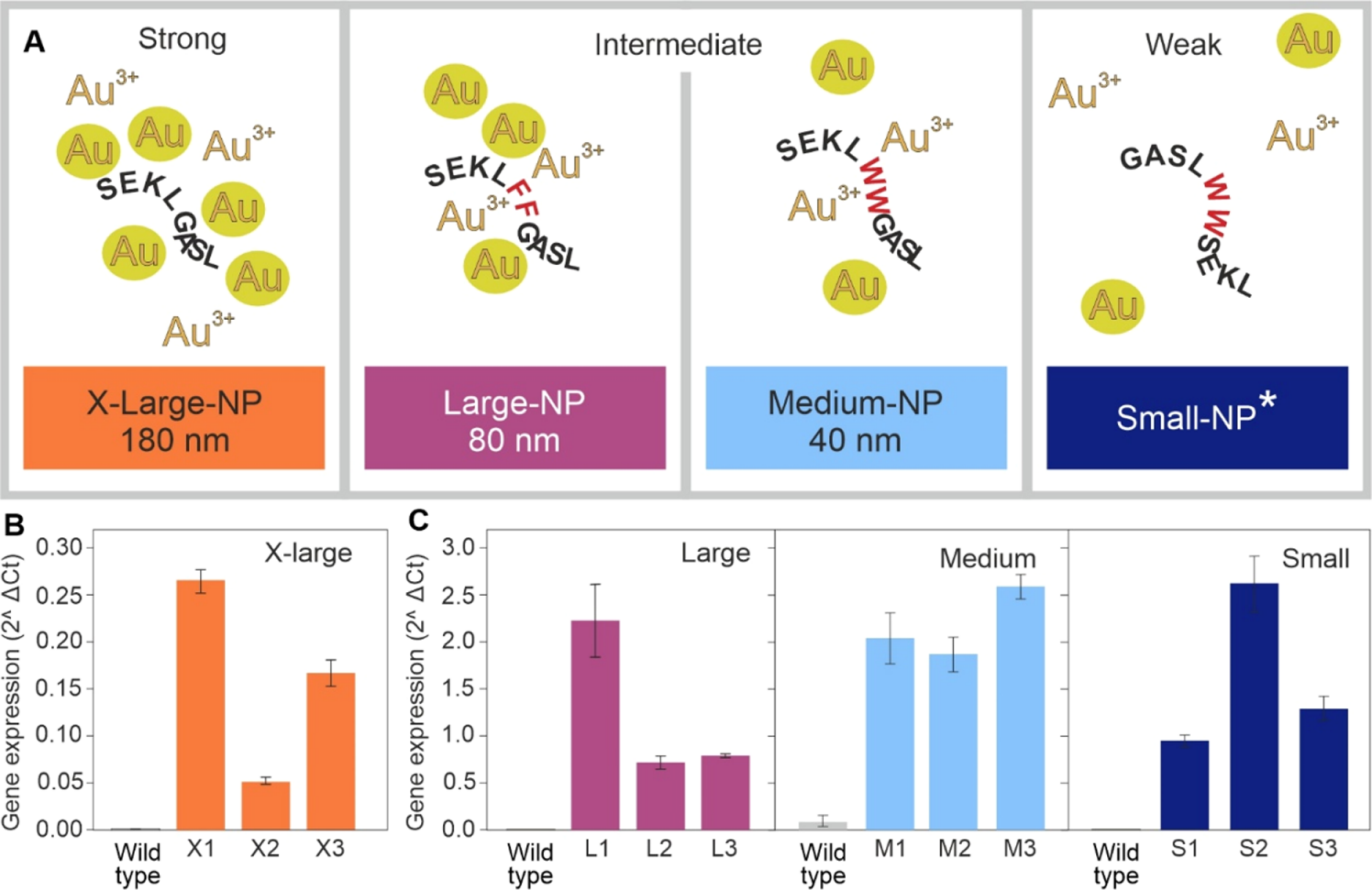
Expressing Au-reducing peptides in *Arabidopsis*. (A) Schematic showing the Au ion reducing and binding properties and the reported NP size from *in vitro* experiments on four synthetic peptide sequences reported by Tan et al.^19^ *, Tan reported that NPs were not observed for *in vitro* studies using this peptide. (B, C) Relative expression of peptide-encoding genes in leaf tissues from plant lines. Fold increase, relative to the *ArabidopsisACTIN* gene, was calculated using the 2∧ΔCt method.^28^ Results are the mean from three biological replicates ± SD; results for three independently transformed plant lines for each peptide-expressing construct are shown. X_n_, L_n_, M_n_, and S_n_ represent results from each of three, independently transformed lines expressing peptide sequences designed to produce X-Large-, Large-, Medium-, and Small-NPs, respectively.

To investigate the combined effect of the expression of the Small-NP peptide alongside increased COPT2 expression, pMLBART-COPT2 was introduced into the previously characterized *Arabidopsis* S1 line (Figure 1C). Three T3 lines homozygous for COPT2 were selected, as described above, and used for further analysis.

### Plant Liquid Culture Experiments

**Figure 2:**
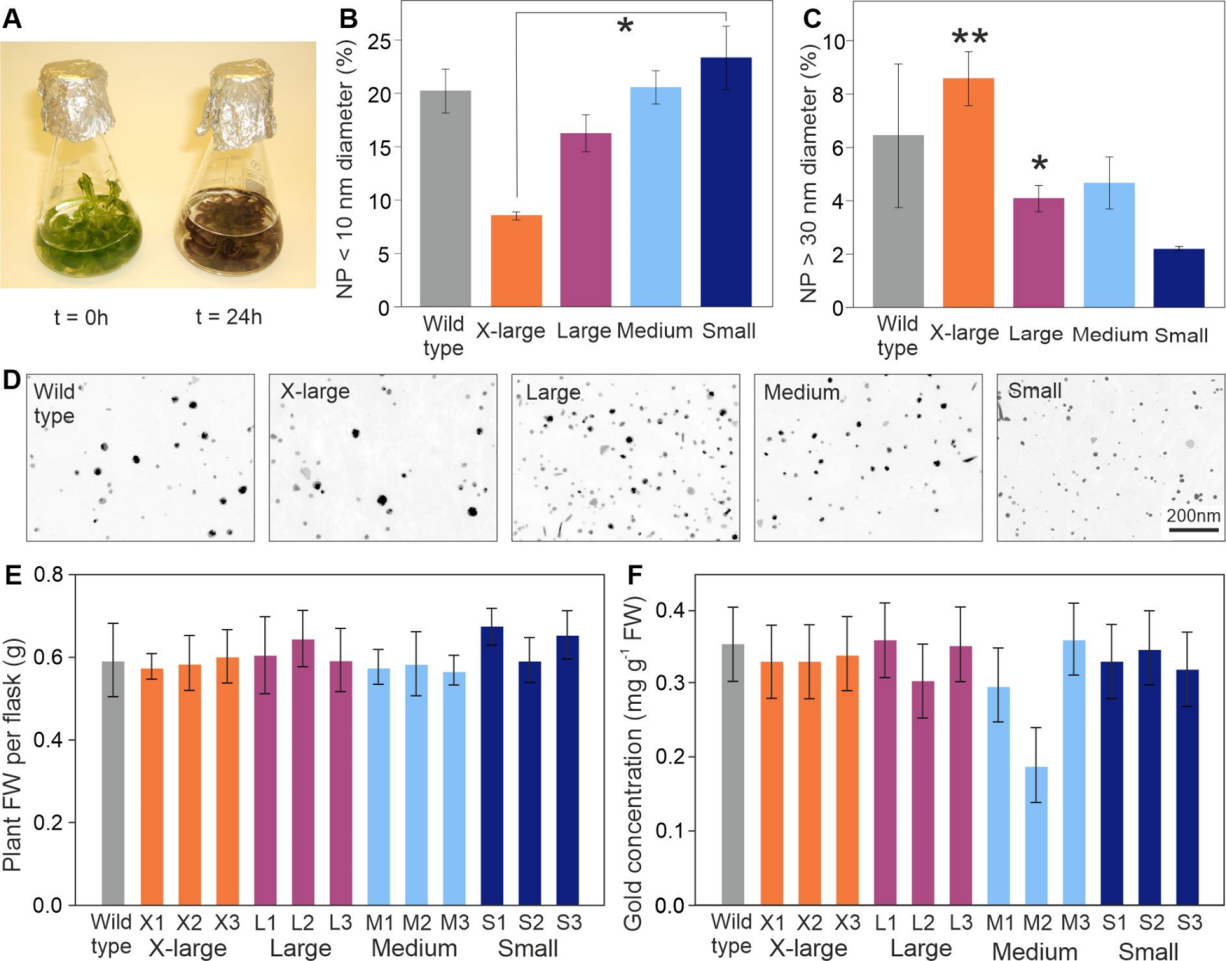
Characterization of Au-NP deposition in peptide-expressing *Arabidopsis*. (A) Appearance of three-week-old, liquid-culture-grown plants immediately prior to dosing and 24 h after dosing with 0.75 mM potassium(III) tetrachloroaurate. (B, C) Percentage distribution of NP diameters in liquid-culture-grown *Arabidopsis* plants expressing synthetic peptides. Results are the mean from three TEM sections from each of three, independently transformed lines for each peptide-expressing construct ± SD; asterisks indicate the level of significant difference (* = *p* < 0.05; ** = *p* < 0.01; *** = *p* < 0.001) tested by one-way ANOVA. (D) Representative transmission electron micrographs showing NPs present in the plastids. (E) Fresh weight (FW) of the plants when harvested 24 h postdosing. (F) Concentration of Au in the plant tissue, determined using ICP-OES.

Sterilized *Arabidopsis* seeds were sprinkled on agar plates containing 1/2 MS medium^30^, stratified in the dark at 4 °C for 3 days, and then seedlings were grown vertically for 7 days. Subsequent liquid culture flasks were set up as described^29^ and are shown in Figure 2A. The plants were grown for 2 weeks, then the medium was replaced with 20 mL of 0.75 mM potassium(III) tetrachloroaurate (KAuCl_4_) in water and returned to the growth room at 130 rpm for a further 24 h.^29^ The whole plant tissue was washed three times in distilled water and then prepared for transmission electron microscopy (TEM), inductively coupled plasma optical emission spectroscopy (ICP-OES), and pyrolysis (see below).

### Plant Hydroponic Experiments

To quantify root:shoot metal partitioning, plants were grown using a hydroponic system. Ten-day-old seedlings, germinated and grown as described above, were transferred into cylindrical sponges placed in polystyrene rafts floating in plastic boxes containing 200 mL of 1/2 MS medium, as shown in Figure 4A. The boxes were placed in a Sanyo growth chamber, with day and night temperatures of 21 and 18 °C, respectively, a 12 h photoperiod, and a 300 μmol·m^2^·s^–1^ light intensity. The growth medium was replaced weekly. Five-week-old plants were dosed with 1 mM KAuCl_4_ and harvested after 24 h of exposure.

### TEM Analysis

Leaf sections were cut under a fixative, then prepared as described.^14^ TEM analysis was conducted using a Tecnai TEM–FEI 12 Bio Twin instrument operating at 120 kV. The NP diameter was measured from TEM images using ImageJ software (version 1.5). A minimum of 100 (but often >250) NPs was measured for each material.

### ICP-OES Analysis

All tissue samples were washed three times with 50 mL of water, and additionally, root tissues were in root desorption solution (2 mM CaSO_4_ and 10 mM ethylenediamine tetraacetic acid) for 10 min at 180 rpm. All tissues were then rinsed three times in water. Samples were oven-dried at 90 °C for 72 h, then 0.1 g of dried tissue was disrupted using a glass rod. The tissue was then sonicated for 20 min in a glass vial containing 10 mL of methanol. Following the evaporation of methanol, samples were digested for 16 h in 500 μL of aqua regia at 110 °C. Cooled samples were diluted with water to a final volume of 10 mL, filtered using glass microfibre filters (Whatman), and then the metal content was determined using an iCAP 7000 series (Thermo Fisher, U.K.). Standards were prepared from Periodic table mix 1 and Periodic table mix 2 (Merck, U.K.), Au was measured at 267.6 nm (axial), and Cu and Fe were measured at 324.8 and 239.6 nm (radial), respectively.

### Formation and Analysis of the Biomass-Derived Catalyst

To produce the catalyst, the plant biomass was air-dried, ground to a powder, and then pyrolyzed using a Barnstead Thermolyne 6000 furnace. Samples were heated to 200 °C with a ramp rate of 10 °C min^–1^ under a N_2_ atmosphere. The maximum temperature was held for 30 min and then reduced to room temperature and sample weights were recorded.

Prior to further analysis of the catalysts, porosity measurements, using a Micrometrics ASAP 2020 porosimeter under liquid nitrogen and N_2_ as absorbance, estimated a catalyst surface area of ∼3.5 m^2^. g^–1^. Previous studies on a range of Au catalysts predicted surface areas greater than ∼1000 m^2^. g^–1;^^31−33^ thus, the reaction time was increased from the reported 4 h^34^ to 24 h. Catalytic activity of the resultant carbonized powder was assessed by following the oxidation of 1,2-propanediol under basic conditions as follows. To a 45 mL pressure reactor, 12 mmol (0.9 mL) of 1,2-propanediol was added together with 1 equiv of NaOH (12 mmol), 20 mL of distilled water, and 50 mg of the catalyst material. The sealed reactor was then washed three times with oxygen prior to pressurizing with 3 bar of oxygen.^34^ The reactor was then heated to *T* = 60 °C, with stirring at 1000 rpm, and allowed to proceed for 24 h. Reactions were monitored using GC-FID with diethyl succinate as a standard. The cooled reaction products were filtered and then purified by flash column chromatography using pentane as the solvent. The ATR FT-IR was carried out on a Bruker Vertex70 ATR FT-IR instrument fitted with a Specac guest ATR with a germanium ATR element. The resolution was given as 4 cm^–1^.

## Results and Discussion

### Generating Peptide-Expressing Transgenic Plants

*Arabidopsis* lines expressing the synthetic peptide sequences listed in Figure 1A and designed to produce X-Large-NP, Large-NP, Medium-NP, and Small-NP, respectively, were generated. Figure 1B demonstrates that all four, small, peptide-encoding sequences were successfully expressed in *Arabidopsis*; with the Small-NP lines producing 10-fold lower expression levels than the other three lines. However, none of the peptides could be detected in plant samples analyzed using C18 and SCX extraction and either MALDI-TOF mass spectrometry or LC/ESI-MS/MS.

### Formation of Au-NPs

Whole-plant, liquid culture experiments were conducted to investigate the potential of the peptides to seed the formation of Au-NPs *in planta*. The preparation and characterization of the biomass-derived Au catalyst described here^14^ were adapted from previously published work^35^ for the synthesis of Au-NPs. The liquid culture was chosen over a soil-based system as Au could be dosed at a relatively high concentration and in the ionic form, enabling sufficient NP deposition for subsequent analysis.

Figure 2A shows the appearance of the plants prior to dosing. At 24 h postdosing, all plant tissues were a dark purple color, as reported previously,^6^ and seen within 3 h for Pd.^14^ This color is indicative of Au-NP formation, the result of surface plasmon resonance of these NPs causing an absorption of light in the blue-green portion of the spectrum while reflecting red-purple light. Large numbers of NPs could be readily discerned in the TEM images of leaf tissues (Figure S1). Subsequent TEM analysis confirmed the presence of NPs located indiscriminately across organelles and cytoplasmic regions (Figure S1). In agreement with the *in vitro* experiments, the Small-NP lines contained the highest (23%) distribution of small (<10 nm) Au-NPs followed by Medium-NP, Large-NP, and X-Large-NP lines with 20, 20, 16, and 8%, respectively (Figure 2B). A trend in the opposite pattern was observed for large (>30 nm) NPs, with the Small-NP, Medium-NP, wild-type, Large-NP, and X-Large-NP containing distributions of 2, 4, 6, 4, and 8% large (>30 nm) NPs, respectively (Figure 2C). Figure 2D shows representative TEM images of the NPs observed in the chloroplasts of all four lines, and full NP size distribution profiles are shown for all lines in Figures S2–S5. The biomass and Au content of Au-dosed peptide-expressing plants were not significantly higher than those for wild-type plants (Figure 2E,F), and when grown in soil, the transformed plants were physically indistinguishable from untransformed plants.

### Catalysis

**Figure 3:**
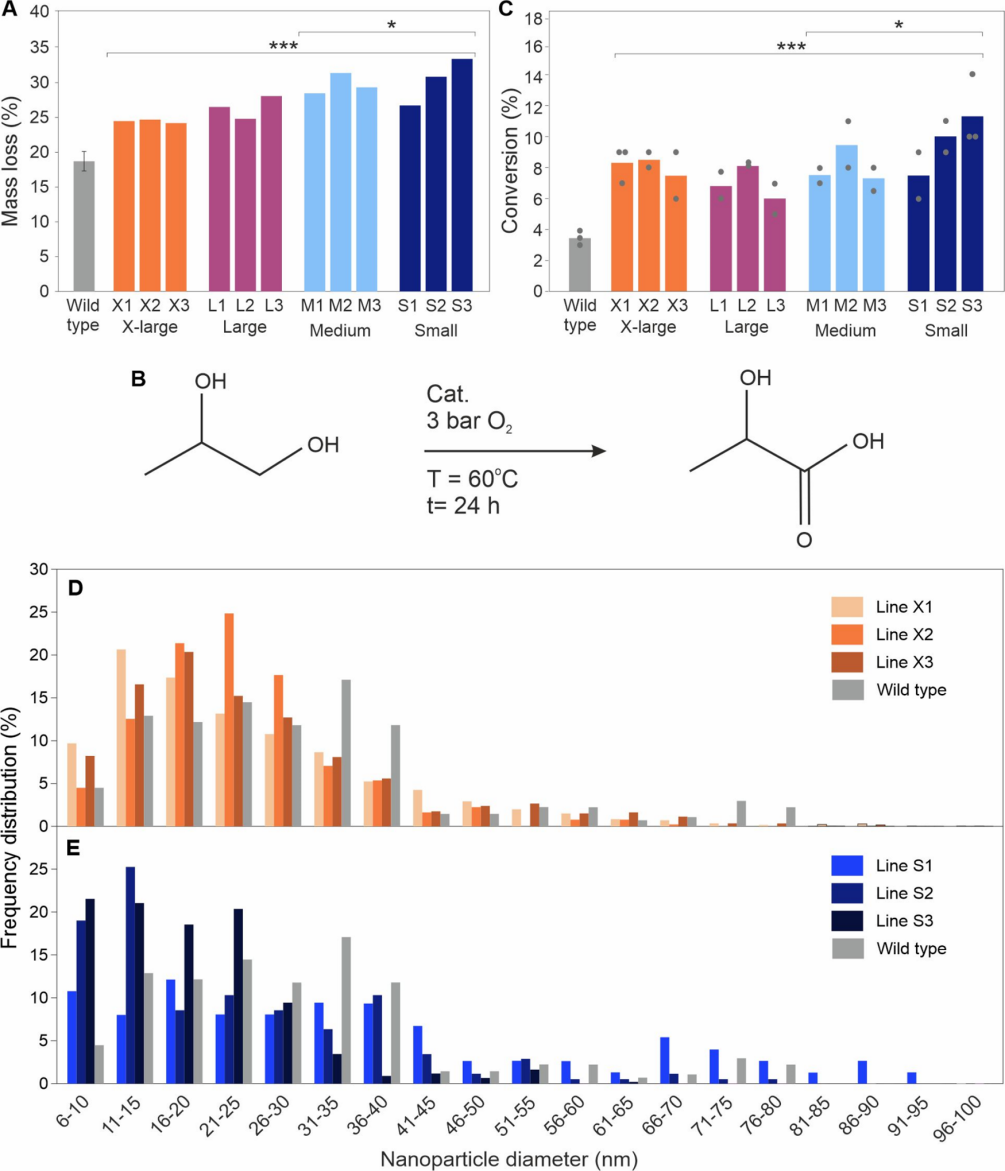
Characterization of the pyrolyzed biomass. (A) Mass loss of the biomass postpyrolysis. Wild type *n* = 3 ± SD, for the transgenic lines; results shown are single measurements for each of three independently transformed lines for each peptide-expressing construct. (B) Reaction scheme for the oxidation of 1,2-propanediol to lactic acid. Mean NP diameter in the pyrolyzed biomass-derived Au material from (C) X-Large- and (D) Small-NP peptide forming lines. (E) Percent conversion of 12 mM 1,2-propanediol to lactic acid under basic conditions. Wild type *n* = 3, transgenic lines, *n* = 2 for each of three independently transformed lines. Asterisks indicate the level of significant difference (* = *p* < 0.05; ** = *p* < 0.01; *** = *p* < 0.001) tested by one-way ANOVA.

Following pyrolysis of the biomass at 200 °C, the mass loss was determined, as shown in Figure 3A. The biomass from wild-type plants exhibited a significantly smaller mass loss, 18.7% ± 1.6, than all of the peptide-expressing lines. While there was only a trend of the increasing mass loss with the decreasing NP size (from X-Large-NP, Large-NP, and Medium-NP to Small-NP lines), the mass loss of the Medium-NP and Small-NP lines was significantly greater than for Large-NP and X-Large-NP lines. Subsequent TEM analysis on the pyrolyzed material showed that relative to the X-Large-NP lines (Figure 3D), the Small-NP lines retained more, smaller NPs postpyrolysis (Figure 3E,). In addition to the smaller NPs, there was also an apparent increase of triangle-shaped NPs in the Small-NP samples (Figure S6) relative to the wild type, but due to their irregular shape, these NPs were difficult to specifically detect with ImageJ and therefore not quantified.

Combining the TEM data with the IR spectra for the Small-NP lines (Figure S7) revealed that the size distribution of the NPs was influenced by the biomass-derived Au catalyst during pyrolysis. The spectrum of the pyrolyzed wild type shows no major changes except a small decrease in the peak in the 3660–3000 cm^–1^ area. In contrast, the Small-NP lines exhibited decreases in the −CH, −OH, −C=O, and −C-O– peaks. These changes could be due to oxidation of the carbonaceous surface or due to coordination to the Au-NPs.

Gold NPs are commonly used in many industrially important reactions including oxidations. Thus, the catalytic activity of the pyrolyzed biomass-derived Au material was tested using the oxidation of 1,2-propanediol to lactic acid (Figure 3B).^34^ Using 1% Au/activated carbon (Au/AC) and relatively mild conditions produced conversions of up to 36%, a reasonable range over which to compare the catalytic activities. Comparing the conversions shown in Figure 3C, all peptide-expressing lines produced significantly higher conversions when compared to those of the wild-type derived material. The conversion percentages for catalysts derived from the biomass from Medium-NP and Small-NP lines were significantly higher (8.1 and 9.9%, respectively) than those derived from Large-NP and X-Large-NP lines. While these conversions may appear low compared to those reported for the Au/AC catalyst,^34^ the Au/AC catalyst is artificially dosed postpyrolysis. Here, the Au-NPs are formed within the plant tissues; thus, some of the NPs are enclosed and potentially not available for the reaction.

The pattern of the catalytic activity is in agreement with the mass loss and NP size distributions. However, the increased activity from the wild type of 57% (line L3) up to 400% (line S3) indicates that this increase is not only due to the potentially higher amount of metal in the samples caused by the higher mass loss during pyrolysis, as line S3 shows just 58% more mass loss than the wild type, a result that we are unable to explain.

### Synthetically Enhancing the Au Uptake Ability

**Figure 4:**
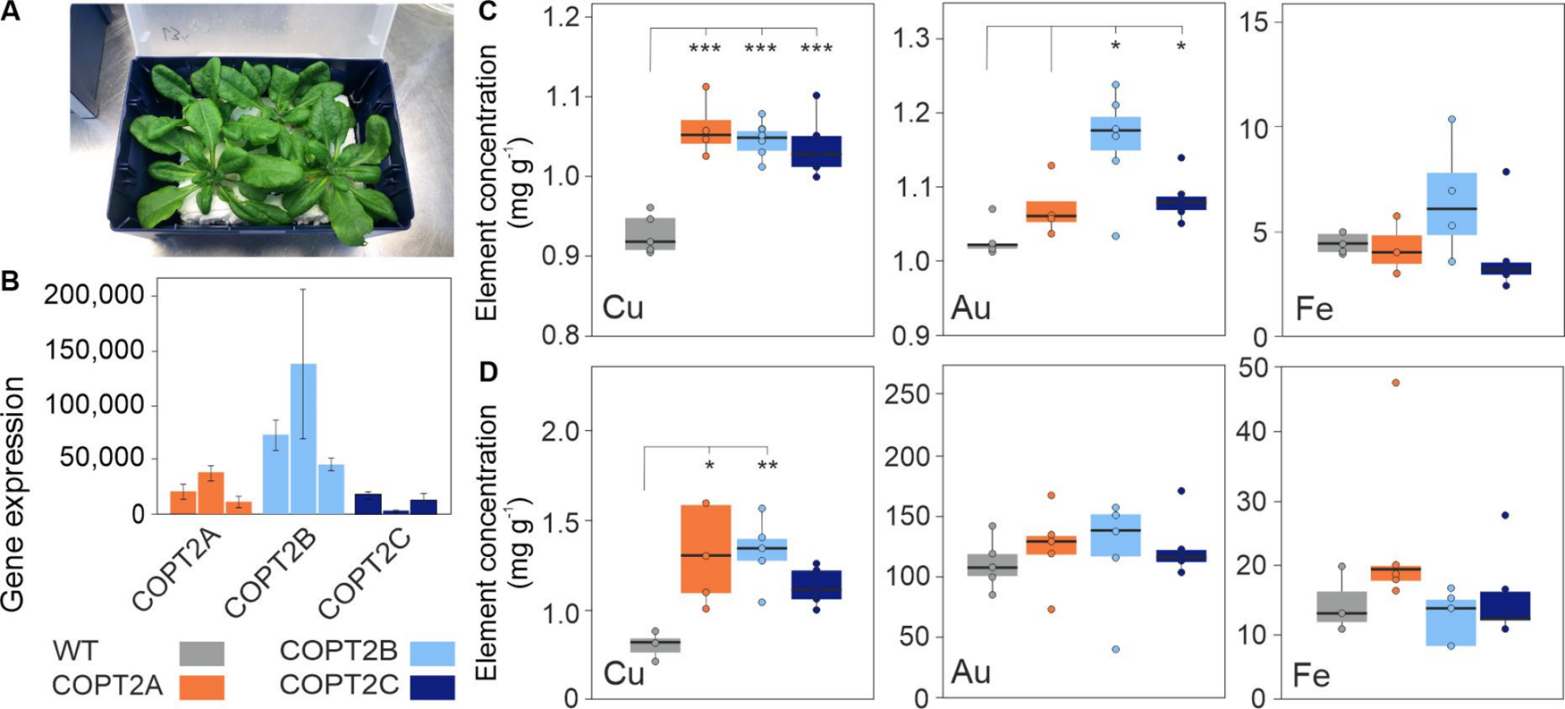
Characterization of 35S-COPT2 only overexpression lines. (A) Appearance of control, five-week-old *Arabidopsis* plants growing hydroponically just prior to dosing. (B) qPCR showing codon-optimized COPT2 transcript abundance in soil-grown *Arabidopsis* rosette leaves for unmodified wild-type (WT) and three independently transformed lines (COPT2A, B, and C). Values were normalized to *ACTIN2* and are means of three biological replicates ± SD for each of three plants per line. Boxplots visualizing Cu; copper, Au; gold, and Fe; iron concentrations in the (C) shoot and (D) root tissue of hydroponically-grown WT and codon-optimized*-*COPT2-expressing lines, 24 h after the addition of 1 mM Au to five-week-old *Arabidopsis*. WT, wild-type; asterisks indicate the level of significant difference (* = *p* < 0.05; ** = *p* < 0.01; *** = *p* < 0.001) tested by one-way ANOVA.

Given the submerged nature of liquid-culture-grown plants, tissue Au concentrations (Figure 2F) are expected to be significantly higher than those achieved in soil-grown systems. Soil-based studies indicate that the uptake of Au is a limiting factor.^6,15^ Research on the Cu transporter (COPT2) has demonstrated that this transporter also takes up Au.^25^ Toward developing a plant-based metal recovery system for Au, we overexpressed COPT2 in *Arabidopsis* and then introduced this trait into the Small-NP peptide-expressing background. Figure 4B shows that codon-optimized COPT2 was expressed in three independently transformed lines. Analysis of codon-optimized COPT2-expressing seedlings showed that under a range of Cu (10 to 40 μM; as CuSO_4_), or Au (50 to 200 μM; as KAuCl_4_), concentrations, root length, the number of lateral root branches, and root architecture were not significantly different to the wild type (results not shown). However, ICP-OES analysis of hydroponically grown plants dosed with 1 mM KAuCl_4_ demonstrated that the codon-optimized, COPT2-expressing lines accumulated significantly more Cu (an element in the MS medium) in aerial tissues than the wild type (Figure 4C); root tissue Cu concentrations were also significantly higher than the wild type for two of the COPT2-expressing lines (Figure 4D). A study on COPT2^25^ suggests that this transporter is more active toward Cu than Au, and in agreement with this, the Au concentrations in the aerial tissues of the COPT2-expressing lines were less pronounced than for Cu. However, two of the lines still accumulated significantly more Au in aerial tissues than the wild type. As expected, given the lack of activity of COPT2 toward iron,^25^ aerial and root tissue iron concentrations were not significantly different to wild-type levels.

**Figure 5:**
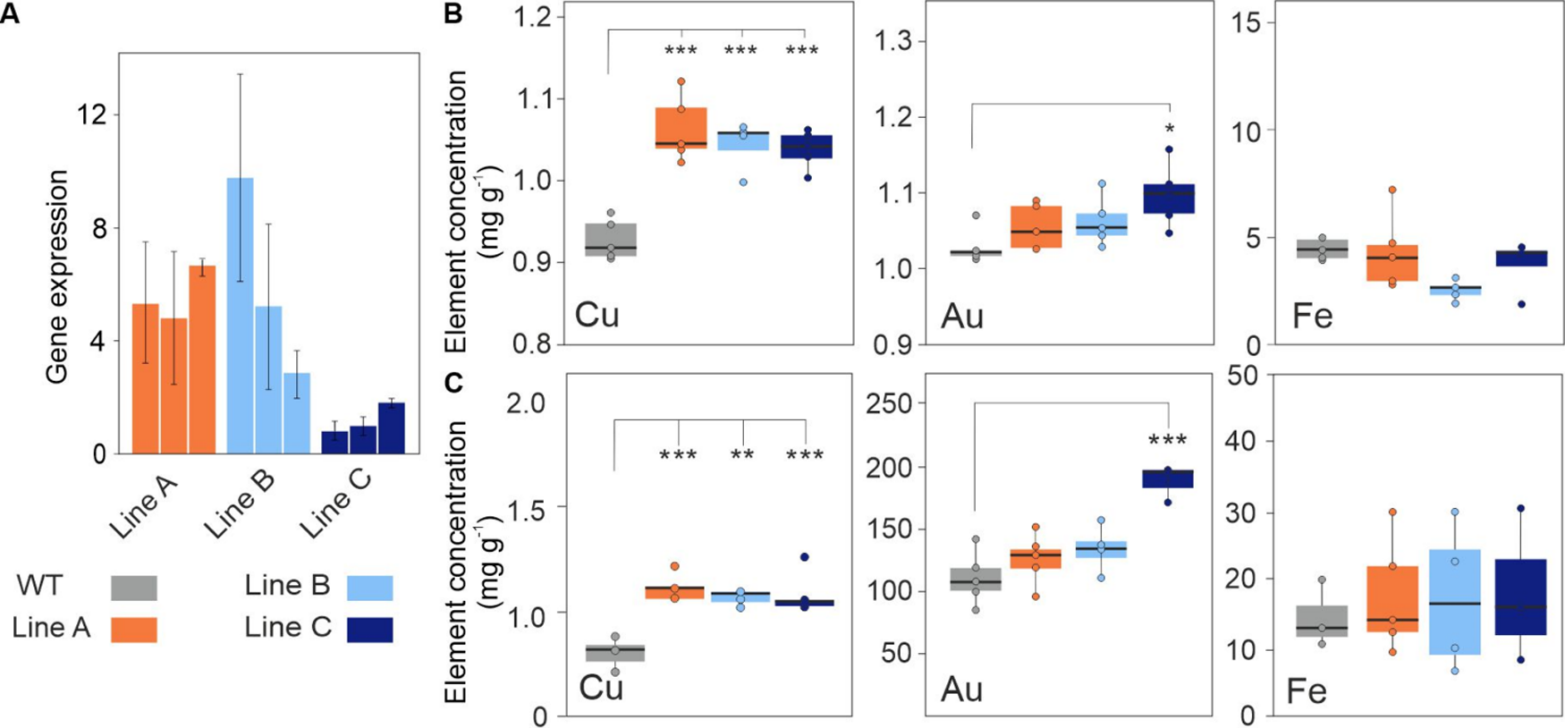
Characterization of double 35S–S1peptide −35S-COPT2 overexpression lines. (A) qPCR showing abundance of the Small-NP peptide transcript in soil-grown rosette leaves of Small-NP*-*COPT2-expressing lines relative to the expression level in the *Arabidopsis* wild type (WT). Values were normalized to *ACTIN2* and are means of three biological replicates ± SD for each of three plants per line. Boxplots visualizing Cu; copper, Au; gold, and Fe; iron concentrations in the (B) shoot and (C) root tissue in hydroponically-grown WT and codon-optimized COPT2 expression lines 24 h after the addition of 1 mM Au to five-week-old *Arabidopsis*. WT, wild type, asterisks indicate the level of significant difference (* = *p* < 0.05; ** = *p* < 0.01; *** = *p* < 0.001) tested by one-way ANOVA.

Having established that the COPT2-expressing lines accumulated more Cu and Au in the shoots, compared to the wild type, the COPT2-expressing construct was transformed into the Small-NP line S1. As shown in Figure 5A, the Small-NP peptide transcript was successfully expressed in the rosette leaves of the Small-NP-COPT2 lines. Hydroponic studies (Figure 5B,C) showed that aerial tissue Cu concentrations in the double Small-NP-COPT2 lines were significantly higher than in the wild-type lines and iron levels were unaltered. However, only one of the three transformed lines exhibited aerial and root Au concentrations that were significantly higher than those in the wild type, and compared to the COPT2-expressing lines shown in Figure 4, Au uptake was not enhanced by upregulating COPT2 in the Small-NP peptide-expressing background.

It has been shown *in vitro* that specific peptide sequences can be used to tune the NP diameter *in vitro*.^19,36^ Here, we have successfully transferred this capability into the plant model system, *Arabidopsis*, by expressing peptide sequences that seed the formation of “small”, “medium”, “large”, and “X-large” NPs *in planta*. This pattern mirrors that seen in the *in vitro* experiments reported by Tan et al.^19^ Furthermore, the pattern is statistically significant: the Small-NP lines accrued significantly more smaller (<10 nm) particles than the X-Large-NP lines, and vice versa. In addition to catalysts, metal NPs can also be used directly in a plethora of industrial, medical, agricultural, and cosmetic applications. However, the use of bioderived NPs for these applications is hindered by their irregular shape and wide range of the NP diameter.^9^ Employing the GM and synthetic biology approaches presented here demonstrates that the Au-NP size can be controlled.^9^ This ability is critical in developing metal NP-containing plant biomass-derived catalysts but also (following purification) for direct use in other applications. Synthetic biology, GM, and nanobiotechnology could also be further employed to effectively tailor NPs for downstream application, controlling NP characteristics and location at the organ and cellular level and reducing the presence of contaminating metals and other elements.

While increasing the expression of COPT2 did increase Au or Cu aerial tissue concentrations, overexpressing COPT2 in combination with the Small-NP peptide did not further increase concentrations of these metals. There are clearly more interactions at play in Au uptake and NP formation, and it is possible that the relatively high (1 mM) concentration of Au in the liquid culture media exceeded the reducing and binding capacities of the peptides present. Using lower Au concentrations and soil-based studies are essential to give more information on *in planta* Au capacity and toxicity thresholds. Undoubtedly, the role of COPT2 in *in planta* application for Au phytomining is still not fully understood.

### Genetic Engineering to Increase the Formation of Metal NPs

Commercial exploitation of phytomining for Au and PGMs is also hindered by the relatively poor solubility and low uptake of these metals.^4,15^ Developing genetic engineering (GM) and plant synthetic biology techniques could overcome these problems. Such approaches have been utilized to improve the ability of plants to accumulate and tolerate heavy metals.^37^ This ability can be achieved by controlling the expression of genes encoding metal sequestration, chelation, or reduction properties. Gene expression studies^6^ have contributed toward an understanding of the mechanisms behind Au uptake and storage. However, the results presented here are proof-of-concept, hydroponic-based studies of the molecular biology model species *Arabidopsis*. If this technology is to be commercialized to recover precious metals from our environment, further studies will be needed to more fully understand plant metal uptake and sequestration and develop methods to transition this technology into biomass crop species.^4^

## Supporting Information Available

Peptide sequences involved in the formation of gold metal NPs and DNA sequences of primers used in cloning and qPCR analysis (Table S1); transmission electron micrographs (TEMs) of *Arabidopsis* tissues after treatment with gold for 24 h (Figure S1); histogram showing the frequency distribution of Au-NPs and TEM images at 105k magnification obtained from *Arabidopsis* wild type and three independently transformed X-Large peptide encoding lines after treatment with gold for 24 h (Figure S2); histogram showing the frequency distribution of Au-NPs and TEM images at 105k magnification obtained from *Arabidopsis* wild type and three independently transformed Large peptide encoding lines after treatment with gold for 24 h (Figure S3); histogram showing the frequency distribution of Au-NPs and TEM images at 105k magnification obtained from *Arabidopsis* wild type and three independently transformed Medium peptide encoding lines after treatment with gold for 24 h (Figure S4); histogram showing the frequency distribution of Au-NPs and TEM images at 105k magnification obtained from *Arabidopsis* wild type and three independently transformed Small peptide encoding lines after treatment with gold for 24 h (Figure S5); transmission electron micrograph images of Au-NPs in pyrolyzed biomass-derived material from wild type and Small-NP-expressing *Arabidopsis* lines (Figure S6); and infrared (IR) spectra for biomass from wild type and Small-NP lines before (black) and after (red) pyrolysis (images are representative traces from 3 replicate IR scans for each line) (Figure S7). (PDF)

## Abbreviations

CCR2: CC chemokine receptor 2
CCL2: CC chemokine ligand 2
CCR5: CC chemokine receptor 5
TLC: thin-layer chromatography
TEM: transmission electron microscopy
ICP-OES: inductively coupled plasma optical emission spectroscopy
qPCR: quantitative real-time PCR
GC-FID: gas chromatography flame ionization detector
ATR FT-IR: attenuated total reflectance Fourier transform infrared spectroscopy
SCX: solid-phase extraction
MALDI TOF: matrix-assisted laser desorption ionization time-of-flight
LC/ESI-MS/MS: liquid chromatography electrospray ionization tandem mass spectrometry

## References

[ref1] Graedel T. E. Grand Challenges in Metal Life Cycles. Nat. Resources Res. 2018, 27 (2), 181–190. 10.1007/s11053-017-9333-8.

[ref2] Graedel T. E.; Harper E. M.; Nassar N. T.; Nuss P.; Reck B. K. Criticality of metals and metalloids. Proc. Natl. Acad. Sci. U.S.A. 2015, 112 (14), 4257–4262. 10.1073/pnas.1500415112. 25831527 PMC4394315

[ref3] Kossoff D.; Dubbin W. E.; Alfredsson M.; Edwards S. J.; Macklin M. G.; Hudson-Edwards K. A. Mine tailings dams: Characteristics, failure, environmental impacts, and remediation. Appl. Geochem. 2014, 51, 229–245. 10.1016/j.apgeochem.2014.09.010.

[ref4] Rylott E. L.; Bruce N. C. Plants to mine metals and remediate land. Science 2022, 377 (6613), 1380–1381. 10.1126/science.abn6337. 36137036

[ref5] Wilson-Corral V.; Anderson C. W. N.; Rodriguez-Lopez M. Gold phytomining. A review of the relevance of this technology to mineral extraction in the 21st century. J. Environ. Manage. 2012, 111 (0), 249–257. 10.1016/j.jenvman.2012.07.037. 22940825

[ref6] Taylor A. F.; Rylott E. L.; Anderson C. W.; Bruce N. C. Investigating the toxicity, uptake, nanoparticle formation and genetic response of plants to gold. PLoS One 2014, 9 (4), e93793 10.1371/journal.pone.0093793. 24736522 PMC3988041

[ref7] Egan-Morriss C.; Kimber R. L.; Powell N. A.; Lloyd J. R. Biotechnological synthesis of Pd-based nanoparticle catalysts. Nanoscale Adv. 2022, 4 (3), 654–679. 10.1039/D1NA00686J. 35224444 PMC8805459

[ref8] Cueva M. E.; Horsfall L. E. The contribution of microbially produced nanoparticles to sustainable development goals. Microbial Biotechnol. 2017, 10 (5), 1212–1215. 10.1111/1751-7915.12788. PMC5609226 28771979

[ref9] Pacardo D. B.; Sethi M.; Jones S. E.; Naik R. R.; Knecht M. R. Biomimetic synthesis of Pd nanocatalysts for the Stille coupling reaction. ACS Nano 2009, 3 (5), 1288–1296. 10.1021/nn9002709. 19422199

[ref10] Kwok R. Inner Workings: How bacteria could help recycle electronic waste. Proc. Natl. Acad. Sci. U.S.A. 2019, 116 (3), 711–713. 10.1073/pnas.1820329116. 30647118 PMC6338871

[ref11] Reeves R. D.; Baker A. J. M.; Jaffré T.; Erskine P. D.; Echevarria G.; van der Ent A. A global database for plants that hyperaccumulate metal and metalloid trace elements. New Phytol. 2018, 218 (2), 407–411. 10.1111/nph.14907. 29139134

[ref12] Nemutandani T.; Dutertre D.; Chimuka L.; Cukrowska E.; Tutu H. The potential of *Berkheya coddii* for phytoextraction of nickel, platinum, and palladium contaminated sites. Toxicol. Environ. Chem. 2006, 88 (2), 175–185. 10.1080/02772240600585842.

[ref13] Hunt A. J.; Anderson C. W. N.; Bruce N.; Munoz García A. M.; Graedel T. E.; Hodson M.; Meech J. A.; Nassar N. T.; Parker H. L.; Rylott E. L.; Sotiriou K.; Zhang Q.; Clark J. H. Phytoextraction as a tool for green chemistry. Green Process. Synth. 2014, 3, 3–22. 10.1515/gps-2013-0103.

[ref14] Parker H. L.; Rylott E. L.; Hunt A. J.; Dodson J. R.; Taylor A. F.; Bruce N. C.; Clark J. H. Supported palladium nanoparticles synthesized by living plants as a catalyst for Suzuki-Miyaura reactions. PLoS One 2014, 9 (1), e87192 10.1371/journal.pone.0087192. 24489869 PMC3906157

[ref15] Harumain Z. A. S.; Parker H. L.; Munoz Garcia A.; Austin M. J.; McElroy C. R.; Hunt A. J.; Clark J. H.; Meech J. A.; Anderson C. W.; Ciacci L.; Graedel T. E.; Bruce N. C.; Rylott E. L. Toward Financially Viable Phytoextraction and Production of Plant-Based Palladium Catalysts. Environ. Sci. Technol. 2017, 51 (5), 2992–3000. 10.1021/acs.est.6b04821. 28191957

[ref16] Doroshenko A.; Budarin V.; McElroy R.; Hunt A. J.; Rylott E.; Anderson C.; Waterland M.; Clark J. Using *in vivo* nickel to direct the pyrolysis of hyperaccumulator plant biomass. Green Chem. 2019, 21 (6), 1236–1240. 10.1039/C8GC03015D.

[ref17] Johar P.; Rylott E. L.; McElroy C. R.; Matharu A. S.; Clark J. H. Phytocat – a bio-derived Ni catalyst for rapid de-polymerization of polystyrene using a synergistic approach. Green Chem. 2021, 23 (2), 808–814. 10.1039/D0GC03808C.

[ref18] Brown S.; Sarikaya M.; Johnson E. A genetic analysis of crystal growth. J. Mol. Biol. 2000, 299 (3), 725–735. 10.1006/jmbi.2000.3682. 10835280

[ref19] Tan Y. N.; Lee J. Y.; Wang D. I. Uncovering the design rules for peptide synthesis of metal nanoparticles. J. Am. Chem. Soc. 2010, 132 (16), 5677–5686. 10.1021/ja907454f. 20355728

[ref20] Ishida T.; Murayama T.; Taketoshi A.; Haruta M. Importance of size and contact structure of gold nanoparticles for the genesis of unique catalytic processes. Chem. Rev. 2020, 120 (2), 464–525. 10.1021/acs.chemrev.9b00551. 31820953

[ref21] Villa A.; Dimitratos N.; Chan-Thaw C. E.; Hammond C.; Veith G. M.; Wang D.; Manzoli M.; Prati L.; Hutchings G. J. Characterisation of gold catalysts. Chem. Soc. Rev. 2016, 45 (18), 4953–4994. 10.1039/C5CS00350D. 27200435

[ref22] Kim J.; Rheem Y.; Yoo B.; Chong Y.; Bozhilov K. N.; Kim D.; Sadowsky M. J.; Hur H. G.; Myung N. V. Peptide-mediated shape- and size-tunable synthesis of gold nanostructures. Acta Biomater. 2010, 6 (7), 2681–2689. 10.1016/j.actbio.2010.01.019. 20083240

[ref23] Hvolbæk B.; Janssens T. V. W.; Clausen B. S.; Falsig H.; Christensen C. H.; Nørskov J. K. Catalytic activity of Au nanoparticles. Nano Today 2007, 2 (4), 14–18. 10.1016/S1748-0132(07)70113-5.

[ref24] Sanz A.; Pike S.; Khan M. A.; Carrió-Seguí À.; Mendoza-Cózatl D. G.; Peñarrubia L.; Gassmann W. Copper uptake mechanism of *Arabidopsis thaliana* high-affinity COPT transporters. Protoplasma 2019, 256 (1), 161–170. 10.1007/s00709-018-1286-1. 30043153

[ref25] Tiwari M.; Venkatachalam P.; Penarrubia L.; Sahi S. V. COPT2, a plasma membrane located copper transporter, is involved in the uptake of Au in Arabidopsis. Sci. Rep. 2017, 7 (1), 11430 10.1038/s41598-017-11896-5. 28900233 PMC5595958

[ref26] Gleave A. P. A versatile binary vector system with a T-DNA organisational structure conducive to efficient integration of cloned DNA into the plant genome. Plant Mol. Biol. 1992, 20 (6), 1203–1207. 10.1007/BF00028910. 1463857

[ref27] Clough S. J.; Bent A. F. Floral dip: a simplified method for Agrobacterium-mediated transformation of *Arabidopsis thaliana*. Plant J. 1998, 16 (6), 735–743. 10.1046/j.1365-313x.1998.00343.x. 10069079

[ref28] Livak K. J.; Schmittgen T. D. Analysis of relative gene expression data using real-time quantitative PCR and the 2(-Delta Delta C(T)) Method. Methods 2001, 25 (4), 402–408. 10.1006/meth.2001.1262. 11846609

[ref29] Tzafestas K.; Razalan M. M.; Gyulev I.; Mazari A. M.; Mannervik B.; Rylott E. L.; Bruce N. C. Expression of a Drosophila glutathione transferase in Arabidopsis confers the ability to detoxify the environmental pollutant, and explosive, 2,4,6-trinitrotoluene. New Phytol 2017, 214 (1), 294–303. 10.1111/nph.14326. 27924627

[ref30] Murashige T.; Skoog F. A revised medium for rapid growth and bioassay with tobacco tissue cultures. Physiol.Plant. 1962, 15, 473–496. 10.1111/j.1399-3054.1962.tb08052.x.

[ref31] Carbon (Nano)materials for Catalysis. In Nanostructured Carbon Materials for Catalysis; Philippe Serp B. M., Ed.; The Royal Society of Chemistry, 2015; Chapter 1, pp 1–45.

[ref32] Cecen K.-O. F. Activated Carbon; Wiley, 2014.

[ref33] Yeganeh M. M.; Kaghazchi T.; Kaghazchi T.; Soleimani M. Effect of Raw Materials on Properties of Activated Carbons. Chem. Eng. Technol. 2006, 29, 1247–1251. 10.1002/ceat.200500298.

[ref34] Ryabenkova Y.; He Q.; Miedziak P. J.; Dummer N. F.; Taylor S. H.; Carley A. F.; Morgan D. J.; Dimitratos N.; Willock D. J.; Bethell D.; Knight D. W.; Chadwick D.; Kiely C. J.; Hutchings G. J. The selective oxidation of 1,2-propanediol to lactic acid using mild conditions and gold-based nanoparticulate catalysts. Catal. Today 2013, 203, 139–145. 10.1016/j.cattod.2012.05.037.

[ref35] Sharma N. C.; Sahi S. V.; Nath S.; Parsons J. G.; Gardea-Torresdey J. L.; Pal T. Synthesis of plant-mediated gold nanoparticles and catalytic role of biomatrix-embedded nanomaterials. Environ. Sci. Technol. 2007, 41 (14), 5137–5142. 10.1021/es062929a. 17711235 PMC2518977

[ref36] Coppage R.; Slocik J. M.; Briggs B. D.; Frenkel A. I.; Naik R. R.; Knecht M. R. Determining peptide sequence effects that control the size, structure, and function of nanoparticles. ACS Nano 2012, 6 (2), 1625–1636. 10.1021/nn204600d. 22276921

[ref37] Rylott E. L.; Bruce N. C. How synthetic biology can help bioremediation. Curr. Opin. Chem. Biol. 2020, 58, 86–95. 10.1016/j.cbpa.2020.07.004. 32805454

