# Supplementary material for: Controlling In Planta Gold Nanoparticle Synthesis and Size for Catalysis
Source: Environ Sci Technol. 2024 May 23;58(22):9714–22. doi: 10.1021/acs.est.4c00266 (PMC11155235; doi:10.1021/acs.est.4c00266)
Supplement: Supplementary file 1 — es4c00266_si_001.pdf [file es4c00266_si_001.pdf]

Controlling *in planta* gold nanoparticle synthesis and size for catalysis

Marc Loskarn[2†], Zakuan A. S. Harumain[13†], Jessica A. Dobson[1], Andrew J. Hunt[2,4], Con Robert McElroy[2], Evaldas Klumbys[1], Emily Johnston[1], Juliana Sanchez Alponti[1], James H. Clark[2], Frans JM. Maathuis[1], Neil C. Bruce[1], Elizabeth L. Rylott[1]**

Pages = 10

Figures = 7

Tables = 1

Supplementary Information

Peptide/ Gene	NP size class	Primer type	Forward primer sequence (5' to 3')	Reverse primer sequence (5' to 3')
SEKLGASL	X-large	cloning	TTTGGAGAGGACACGCTCGAGTGTCGCTATGTCTGAAAAGTTGGGAGCTTCTCTTTAATGACGATCATATGAAGCTTTCGAGGAATTCGGTA	TACCGAATTCCTCGAAAGCTTCATATGATCGTCATTAAAGAGAAGCTCCCAACTTTTCAGACATAGCGACACTCGAGCGTGTCCTCTCCAAA
		qPCR	ATGTCTGAAAAGTTGGGAG	Universal reverse primer
SEKLFFGASL	Large	cloning	TTTGGAGAGGACACGCTCGAGTGTCGCTATGTCTGAAAAGTTGTTTTTGGAGCTTCTCTTTAATGACGATCATATGAAGCTTTCGAGGAATTCGGTA	TACCGAATTCCTCGAAAGCTTCATATGATCGTCATTAAAGAGAAGCTCCAAAAAACAACTTTTCAGACATAGCGACACTCGAGCGTGTCCTCTCCAAA
		qPCR	ATGTCTGAAAAGTTGTTTTTTGG	Universal reverse primer
SEKLWWGASL	Medium	cloning	TTTGGAGAGGACACGCTCGAGTGTCGCTATGTCTGAAAAGTTGTGGTGGGGAGCTTCTCTTTAATGACGATCATATGAAGCTTTCGAGGAATTCGGTA	TACCGAATTCCTCGAAAGCTTCATATGATCGTCATTAAAGAGAAGCTCCCCACCACAACTTTTCAGACATAGCGACACTCGAGCGTGTCCTCTCCAAA
		qPCR	TCTGAAAAGTTGTGGTGGGGA	Universal reverse primer
GASLWWSEKL	Small	cloning	TTTGGAGAGGACACGCTCGAGTGTCGCTATGGGAGCTTCTCTTTGGTGGTCTGAAAAGTTGTAATGACGATCATATGAAGCTTTCGAGGAATTCGGTA	TACCGAATTCCTCGAAAGCTTCATATGATCGTCATTACAACTTTTCAGACCACCAAAGAGAAGCTCCCATAGCGACACTCGAGCGTGTCCTCTCCAAA

		qPCR	GGTGGTCTGAAAAGTT GTAATGA	Universal reverse primer
pART7	-	PCR	ACGTTCCAACCACGTCT T	GAATGAACCGAAACCGG CG
ACTIN2		qPCR	TACAGTGTCTGGATCG GTGGTT	CGGCCTTGGAGATCCAC AT
COPT2	-	qPCR	CCTACGTGTCAGTGGCT CAA	GGACATAACAGCGAGCA TCA

Supplementary Table 1. Peptide sequences involved in formation of gold metal NPs, and DNA sequences of primers used in cloning and qPCR analysis.

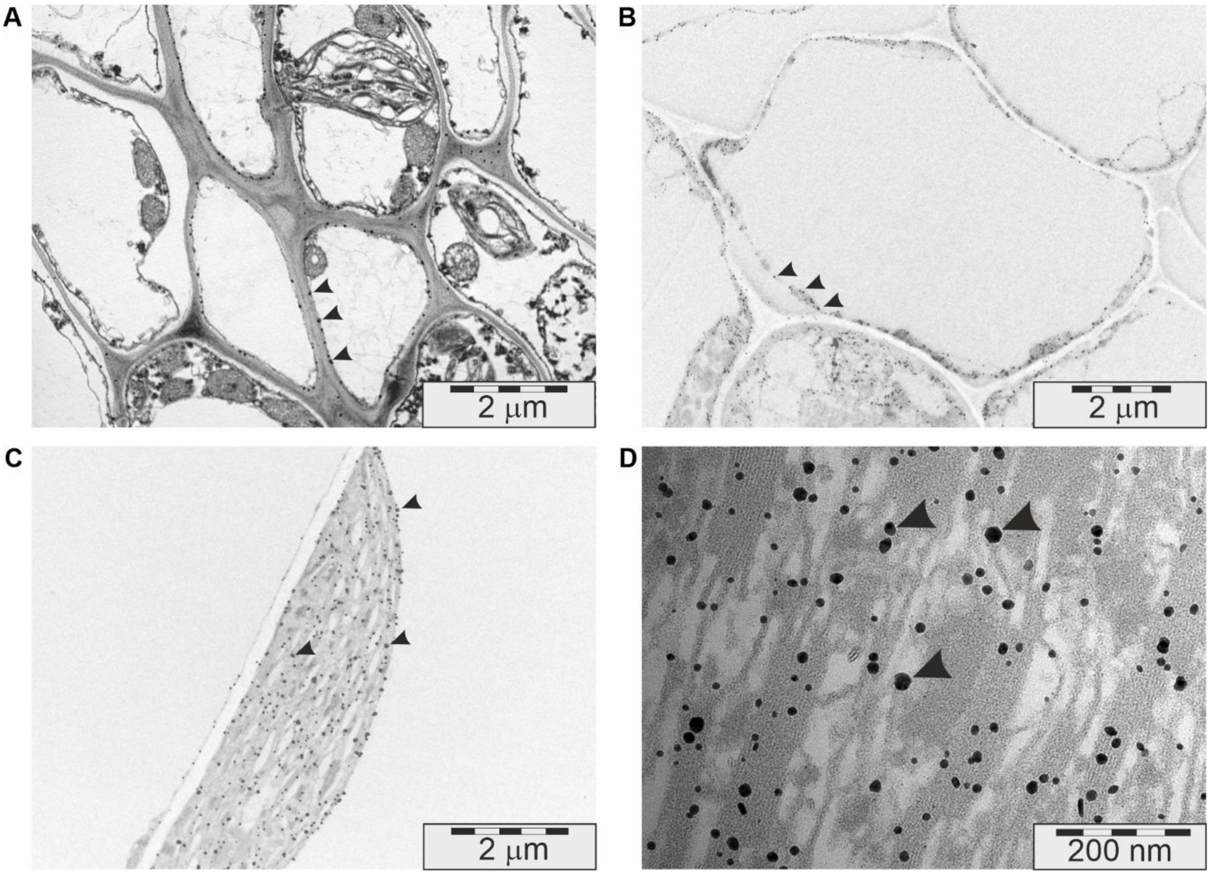

Figure S1. Transmission electron micrographs (TEMs) of Arabidopsis tissues after treatment with gold for 24 hours. **A**) leaf vascular bundle, **B**) mesophyll cell, **C**) chloroplast, **D**) thylakoid membranes. Arrows indicate Au-NPs.

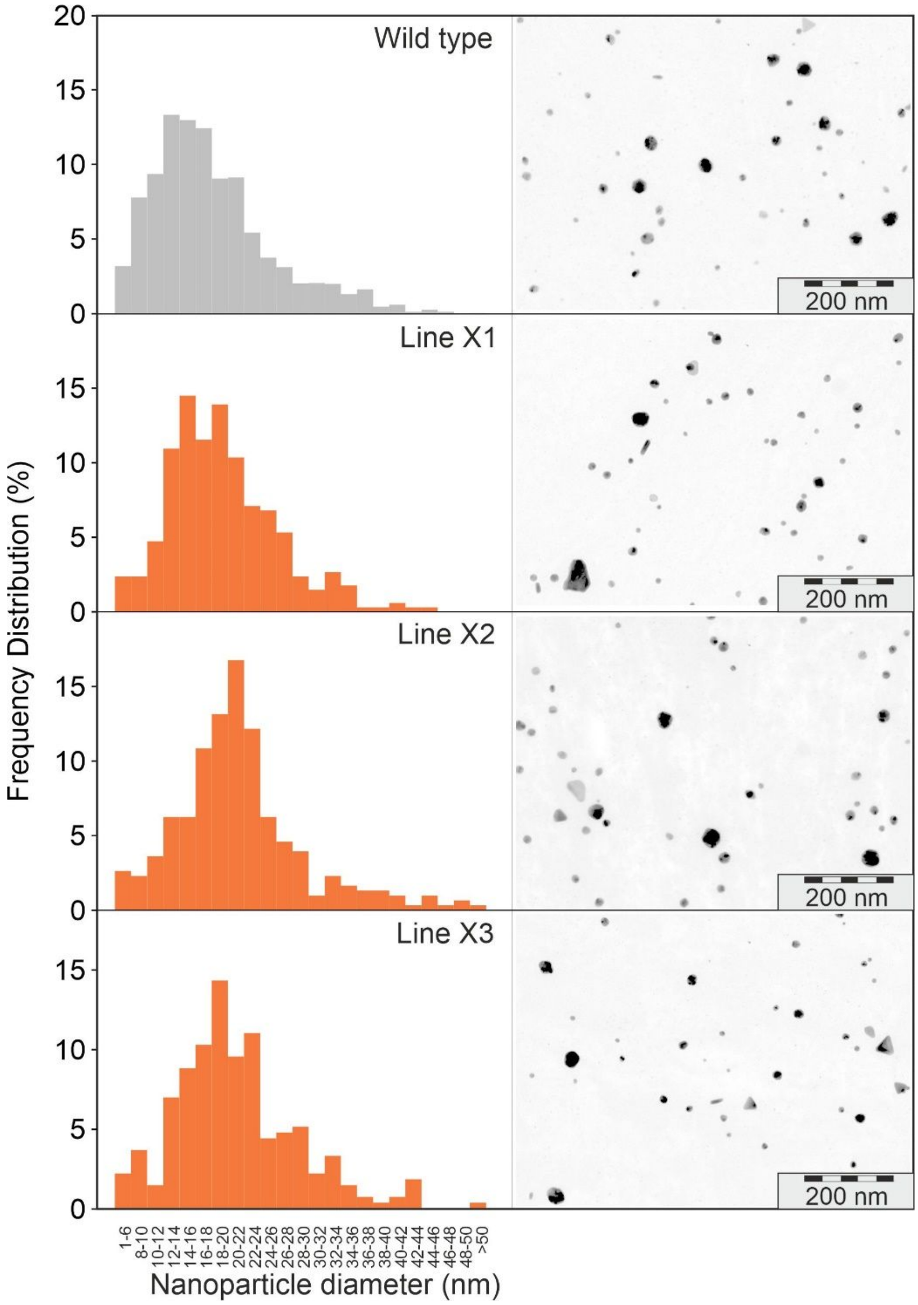

Figure S2. Histogram showing frequency distribution of Au-NPs and TEM images at 105k magnification obtained from Arabidopsis wild type and three, independently-transformed X-large peptide encoding lines after treatment with gold for 24 hours. Results are from a single leaf for each line.

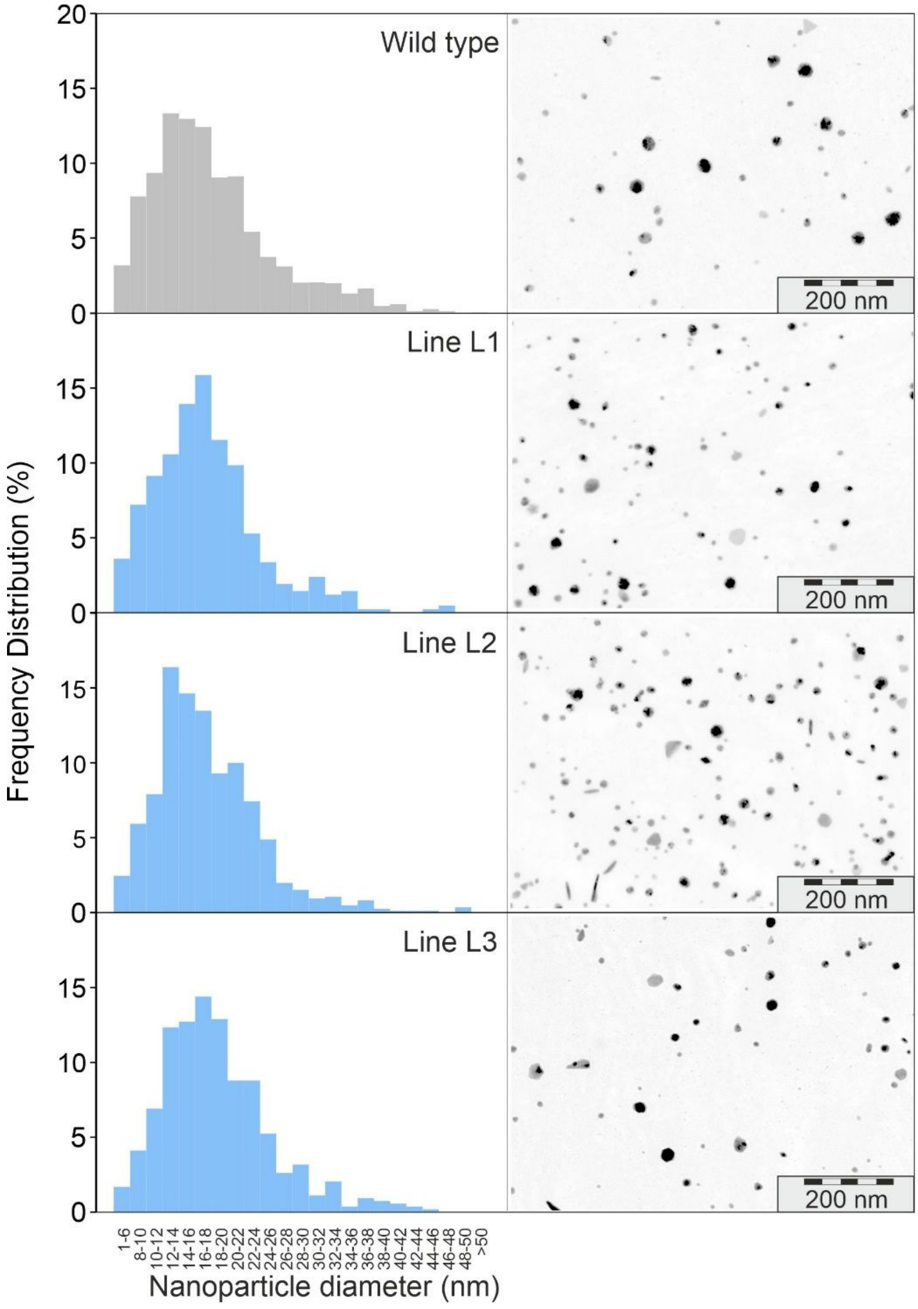

Figure S3. Histogram showing frequency distribution of Au-NPs and TEM images at 105k magnification obtained from Arabidopsis wild type and three, independently-transformed Large peptide encoding lines after treatment with gold for 24 hours. Results are from a single leaf for each line.

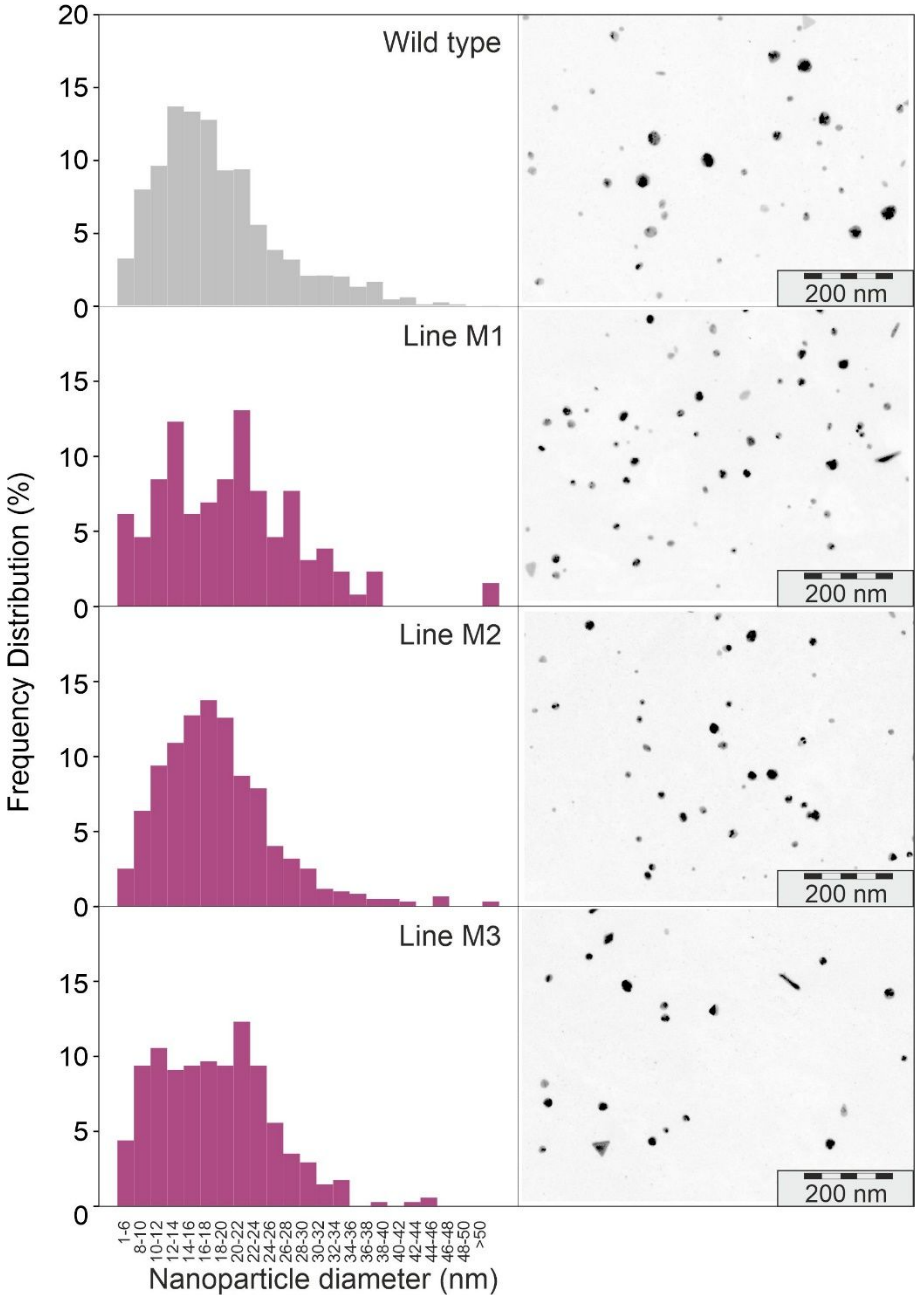

Figure S4. Histogram showing frequency distribution of Au-NPs and TEM images at 105k magnification obtained from Arabidopsis wild type and three, independently-transformed Medium peptide encoding lines after treatment with gold for 24 hours. Results are from a single leaf for each line.

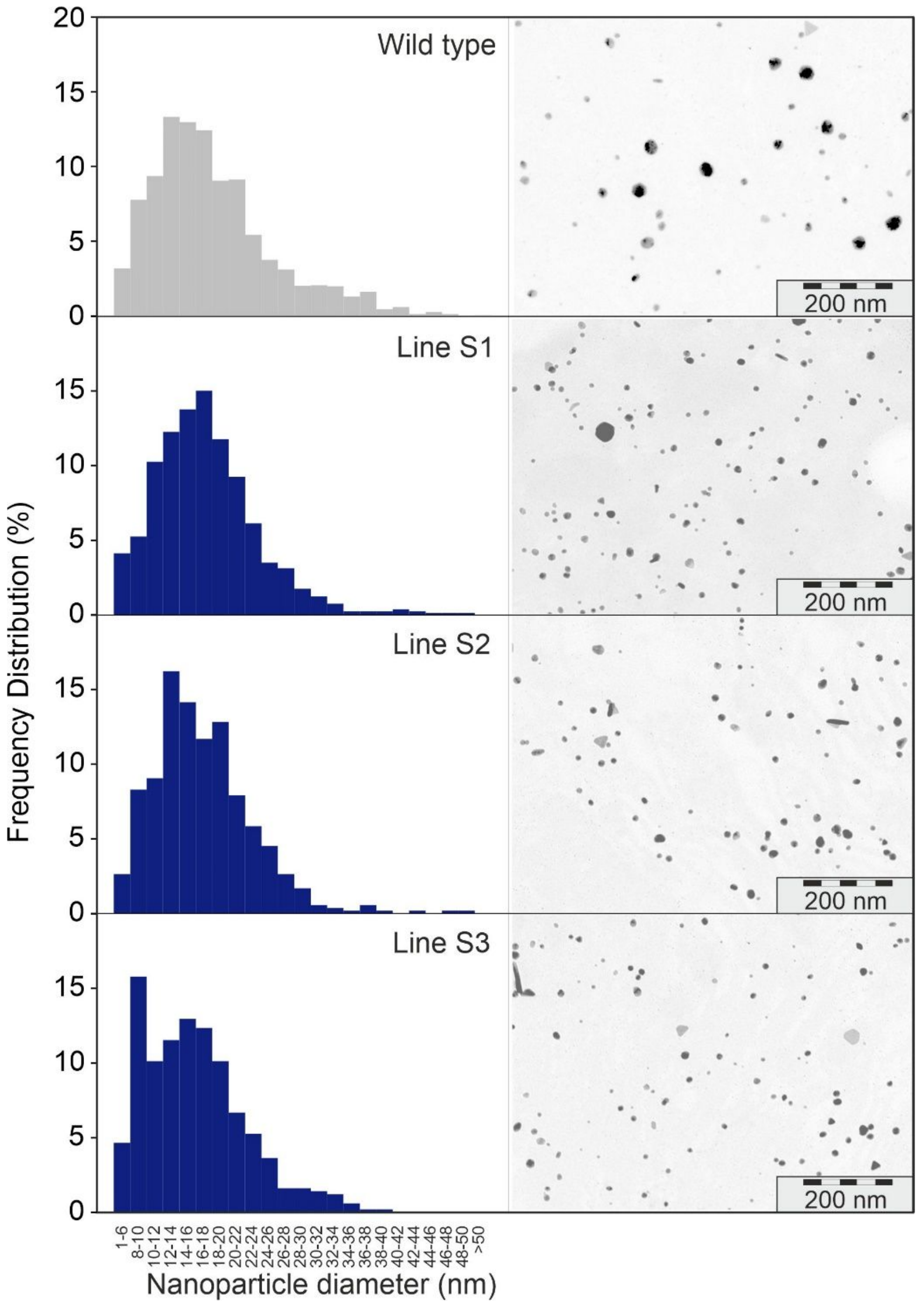

Figure S5. Histogram showing frequency distribution of Au-NPs and TEM images at 105k magnification obtained from Arabidopsis wild type and three, independently-transformed Small peptide encoding lines after treatment with gold for 24 hours. Results are from a single leaf for each line.

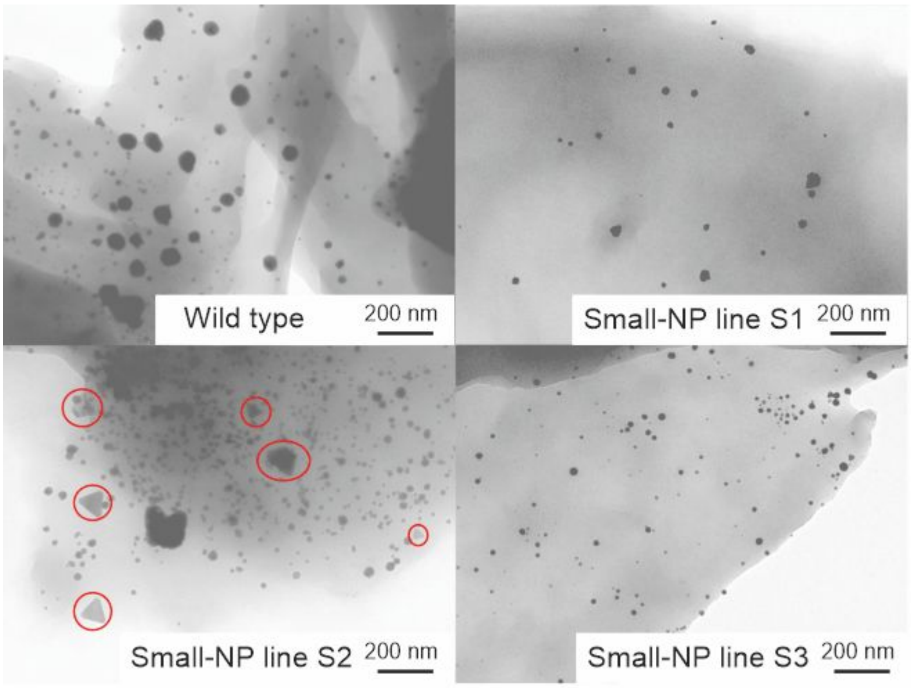

Figure S6. Transmission electron micrograph images of Au-NPs in pyrolysed biomass-derived material from wild type and Small-NP-expressing Arabidopsis lines. Red circles highlight triangular NPs in line 2, but present in all three lines.

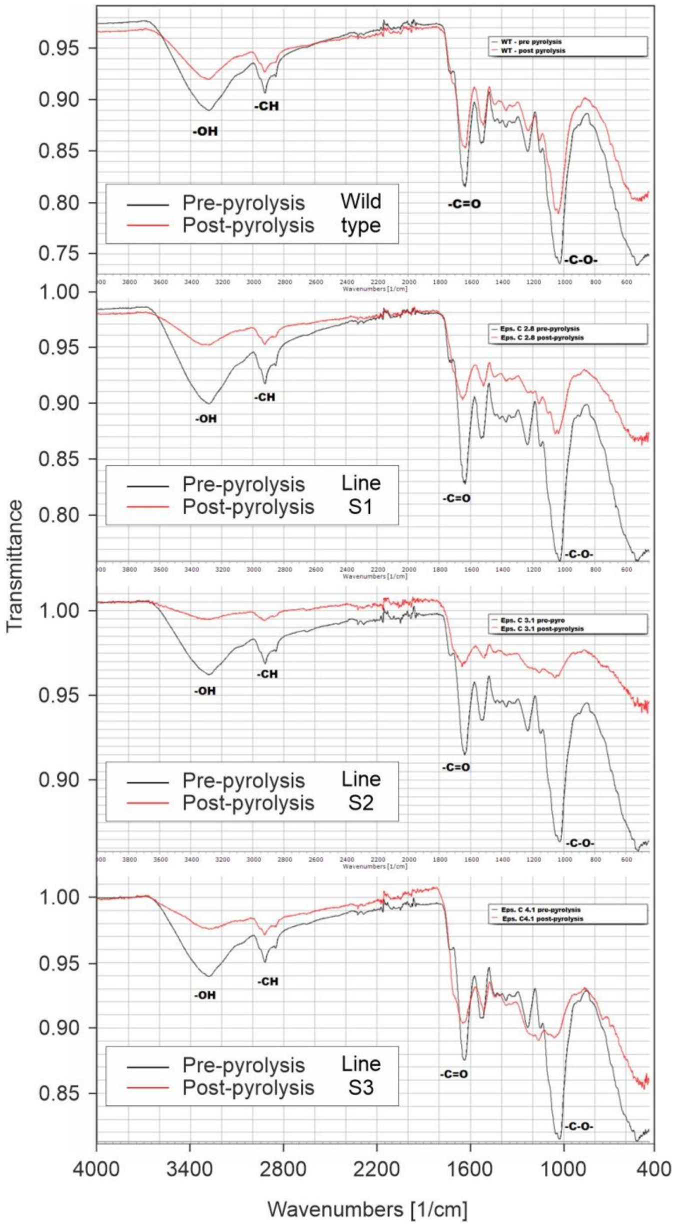

Figure S7. Infra-Red (IR) spectra for biomass from wild type and Small-NP lines before (black) and after pyrolysis (red); images are representative traces from 3 replicate IR scans for ach line.
